# Co-Culture Reveals the Quorum-Sensing Regulatory Mechanism of Bacteriocin PlnJK Synthesis in *Lactiplantibacillus plantarum* EL2

**DOI:** 10.3390/microorganisms14040730

**Published:** 2026-03-24

**Authors:** Fengming Liu, Yixuan Lin, Qi Liang, Xuhui Chen, Baotang Zhao

**Affiliations:** 1 Functional Dairy Products Engineering Laboratory of Gansu Province, College of Food Science and Engineering, Gansu Agricultural University, Lanzhou 730070, China; 2 Agricultural Product Quality and Safety Center of Linxia City, Linxia City 731100, China

**Keywords:** *Lactiplantibacillus plantarum*, bacteriocin, co-culture, quorum sensing, gene expression level

## Abstract

*Lactiplantibacillus plantarum* EL2, isolated from traditional fermented yak milk in the high-altitude Gannan Tibetan Autonomous Prefecture, produces the class IIb bacteriocin PlnJK. This study established three distinct cultivation models that critically influenced bacteriocin yield. Microbial co-culture was found to enhance the stress tolerance of EL2, significantly boosting PlnJK production. The optimal inducing strain, *Enterococcus faecalis* MH2, increased the bacteriocin inhibition zone diameter from 15.38 mm to 25.58 mm. Following optimization of key parameters—initial inoculum concentration (10^7^ CFU/mL), inoculation ratio (3:1, EL2:MH2), and initial pH (6.0)—the inhibition zone diameter reached 30.32 mm, representing a 1.97-fold increase over pure culture. Co-culture not only advanced the onset but also extended the duration of bacteriocin synthesis. Throughout the 24 h incubation, cell density, AI-2 autoinducer concentration, and the expression of key regulatory genes were significantly elevated in co-culture compared to monoculture, aligning with a cell-density-dependent, quorum-sensing (QS) regulatory paradigm. Bacteriocin production was co-regulated by two QS pathways: the AI-2/*luxS* system and the plnA-mediated autoinducing peptide (AIP). Gene expression analysis revealed differential temporal regulation: *luxS* expression was higher during the exponential phase (2.29 vs. 1.42 in stationary phase), while plnA exhibited the opposite pattern (1.42 in exponential vs. 2.21 in stationary phase). This indicates that the AI-2/*luxS* pathway drives strong induction during active growth, whereas plnA/AIP-mediated promotion becomes predominant later. The stationary-phase effect is likely triggered by the accumulation of specific MH2 metabolites, which impose an environmental stress on EL2, stimulating the pln-encoded regulatory system and further enhancing bacteriocin yield. This work provides an economically viable strategy and a novel theoretical framework for optimizing microbial cultivation, enhancing bacteriocin production, and elucidating the complex QS-mediated regulatory mechanisms involved.

## 1. Introduction

Bacteriocins are ribosomally synthesized antimicrobial peptides or proteins produced by lactic acid bacteria (LAB), exhibiting potent activity against foodborne pathogens and spoilage microorganisms. Regarded as safe for human consumption, they are widely recognized as natural food preservatives with broad application potential [1,2,3]. Many LAB strains harbor the genetic capacity to produce bacteriocins [4,5,6,7,8]. However, bacteriocin production is influenced by various factors, including culture conditions, expression of biosynthetic genes, and external stimuli. Although strategies have been developed to modulate these factors, challenges related to safety, yield stability, and genetic consistency remain significant hurdles for industrial application [9].

*L. plantarum* EL2, isolated from traditionally fermented yak milk in the high-altitude Gannan Tibetan Autonomous Prefecture of the eastern Tibetan Plateau, produces the class IIb bacteriocin PlnJK [10]. PlnJK exhibits broad-spectrum activity, inhibiting both Gram-positive bacteria and Gram-negative pathogens such as *S. enterica*. Having evolved in natural fermentation systems with fluctuating temperatures and pH, EL2 demonstrates robust adaptability to environmental instability—a trait that may lower technical barriers and resource demands in fermentation processes. However, under overly extreme culture conditions, bacteriocin production can still decline or cease entirely. Microbial co-culture has been shown to significantly influence and enhance bacteriocin synthesis in lactic acid bacteria [11]. For example, when the non-producer *L. plantarum* J23 was co-cultured with 45 inducing strains, bacteriocin production was activated in 10 of these pairings [12]. Co-culture of *Lactococcus lactis* with *Enterobacter* increased nisin yield by 85% [13], while optimizing the inoculation ratio and incubation time for *Lactobacillus paracasei* HD1.7 with *lacticaseibacillus* markedly boosted the production of bacteriocin Paracin 1.7 [14]. These findings underscore that screening suitable inducing partners and optimizing co-culture conditions can substantially improve bacteriocin titers, highlighting the importance of elucidating the underlying regulatory mechanisms.

Quorum sensing (QS) is a core molecular mechanism that enables microorganisms to achieve intraspecific or interspecific cell-to-cell communication and coordinated regulation of population behaviors through the secretion, recognition, and response to autoinducer signaling molecules. This mechanism can precisely regulate bacteriocin biosynthesis in lactic acid bacteria (LAB) via cascade signaling pathways [15]. In the QS regulatory pathway governing bacteriocin synthesis in LAB, the core regulatory mechanism is the two-component system (TCS). In this system, microorganisms first synthesize and secrete autoinducer (AI) signaling molecules. Once the extracellular concentration of these signaling molecules reaches a threshold level, they are specifically recognized and bound by the histidine protein kinase (HPK) encoded by the *plnB* gene. Upon binding, the HPK undergoes autophosphorylation and subsequently transfers the phosphate group to the response regulator (RR) encoded by either the *plnC* or *plnD* gene, thereby activating it through phosphorylation [16]. The activated response regulator subsequently binds to the promoter regions of downstream target genes, specifically inducing the transcription of functional genes involved in bacteriocin synthesis. These include the *plnJ*, *plnK*, *plnE*, *plnF*, and *plnN* genes, which encode the bacteriocin precursor peptides; the *plnI* gene, which encodes the immunity protein; and the *plnGH* genes, which encode the secretion and transport proteins. Ultimately, this cascade leads to the successful synthesis, self-immunity, and extracellular transport of bacteriocin [17]. Autoinducer signals are primarily categorized into two types: the intraspecific signal peptide AIP encoded by *plnA*, and interspecific signals, namely AI-2, which is produced via the enzymatic activity of the LuxS protein encoded by *luxS* [18,19]. It is generally accepted that in microbial co-culture systems, interspecific QS mediated by AI-2/*luxS* is the predominant factor influencing bacteriocin yield [20]. However, Liu et al. demonstrated through the addition of an AI-2 inhibitor that both AI-2 and AIP still play significant and independent regulatory roles in the process of *Bacillus* BS15 inducing bacteriocin synthesis in *L. plantarum* RX-8 [21]. This question necessitates further investigation into the QS system. Notably, while research on microbial co-culture in the food sector has largely focused on its impact on food quality, studies specifically investigating the underlying QS mechanisms and intercellular signaling molecules remain limited [22]. Consequently, the relationship between the AIP/*plnA* pathway and the AI-2/*luxS* pathway in co-culture systems, as well as their similarities and differences in modulating the two-component system (HPK and RR), remain unclear.

In this study, three distinct cultivation models—simulating harsh (extreme), sub-optimal, and favorable environments—were established to investigate bacteriocin synthesis. By harnessing the inducing potential of microbial co-culture, the production of bacteriocin PlnJK was significantly enhanced under each condition. This strategy presents a practical and economical alternative to the stringent cultivation requirements typical of fermentation processes and provides a conceptual framework for using co-culture to improve bacteriocin yield in lactic acid bacteria (LAB). Furthermore, this work elucidates the mechanisms underlying co-culture-induced high-efficiency bacteriocin synthesis in strain EL2 through multi-level analysis, identifying key genetic determinants. These insights advance the functional understanding of QS-associated genes and offer a technical foundation for the scalable industrial production of LAB bacteriocins.

## 2. Materials and Methods

### 2.1. Experimental Strains

The bacteriocin-producing strain used in this study was *Lactiplantibacillus plantarum* EL2.

The co-culture inducing strains used were *Saccharomyces cerevisiae* SD-1, SD-7; *Pichia dushanensis* BK-11; *Bacillus subtilis* QSM27, QSM199; *Bacillus pumilus* CF88; *Lactiplantibacillus plantarum* EA3, CD11; *Lactococcus lactis* SL1; *Leuconostoc mesenteroides* SL2; *Enterococcus durans* S12; *Enterococcus faecalis* S84, MH2; *Limosilactobacillus fermentum* MK12; *Lacticaseibacillus paracasei* AC13. These strains were originally isolated from distinct ecological niches, including fermented milk, wine, and soil. Preliminary safety assessments, encompassing antibiotic susceptibility and hemolytic activity tests, indicated a favorable safety profile. All strains are currently preserved at the Gansu Provincial Functional Dairy Engineering Laboratory.

The antibacterial indicator strain used in this study was *Salmonella enterica* ATCC 13076.

The AI-2 autoinducer reporter strain used was *Vibrio harveyi* BB170.

Hemolytic control strain: *Staphylococcus aureus*.

### 2.2. Materials

All culture media, including De Man, Rogosa and Sharpe Broth (MRS) Broth/Agar, Yeast Extract Peptone Dextrose (YPD) Broth/Agar, Tryptone Soy Agar (TSA), Bacteriocin Production Medium (BPM), Lysogeny Broth (LB) Broth/Agar, Potato Dextrose Broth (PDB), Brain Heart Infusion Broth (BHI), and Tryptone Soy Broth (TSB), were purchased from Beijing Coolaber Science and Technology Co., Ltd. (Beijing, China).

All chemicals and kits used in this study were obtained from commercial suppliers. Catalase, Proteinase K, and Phosphate-Buffered Saline (PBS) were purchased from Shanghai Yuanye Bio-Technology Co., Ltd. (Shanghai, China). The RNA Rapid Extraction Kit and dialysis tubing were sourced from Beijing Solarbio Science & Technology Co., Ltd. (Beijing, China). Physiological saline, ammonium sulfate, and sodium hydroxide were supplied by China National Pharmaceutical Group Co., Ltd. (Sinopharm, Beijing, China).

### 2.3. Design of Culture Systems for Suboptimal, Low, and Favorable Bacteriocin Production

#### 2.3.1. Design of a Gradient of Cultivation Conditions

With the culture medium (MRS) and incubation time (24 h) held constant, three key factors affecting bacteriocin synthesis—cultivation temperature, initial pH, and inoculum concentration—were varied to establish the experimental conditions detailed in Table 1.

#### 2.3.2. Collection and Preparation of Cell-Free Fermented Supernatant

Under each of the 12 cultivation conditions, activated *L. plantarum* EL2 cultures were inoculated into MRS broth and incubated for 24 h. Subsequently, the cultures were centrifuged (10,000× *g*, 10 min, 4 °C). The resulting supernatant was aseptically filtered through a 0.22 μm pore-size membrane to obtain cell-free supernatant (CFS), following established protocols [23].

#### 2.3.3. Preparation of Crude Bacteriocin

Bacteriocin was partially purified from the CFS of strain EL2 using ammonium sulfate precipitation. Briefly, the CFS was first adjusted to pH 7.0 with 1 M NaOH. Solid ammonium sulfate was then slowly added with constant stirring at 4 °C to achieve 70% saturation. The mixture was incubated overnight at 4 °C to allow for protein precipitation, followed by centrifugation (10,000× *g*, 10 min, 4 °C). The resulting pellet was redissolved in 0.2 mol/L phosphate-buffered saline (PBS, pH 7.0) for subsequent analysis, as described previously [24].

Based on the reported molecular weight of bacteriocin PlnJK (approximately 3–3.5 kDa), a two-step desalting and concentration procedure was performed. The solution was sequentially dialyzed using membranes with molecular weight cutoffs of 4 kDa and 2.5 kDa, followed by lyophilization for 48 h to obtain semi-purified PlnJK [25]. Previous purification and activity profiling within our research group confirmed that the antimicrobial activity in extracts prepared by this method is specific to PlnJK. Therefore, in this study, the antibacterial activity of the crude extract was used as a direct indicator of PlnJK production.

#### 2.3.4. Antibacterial Activity Assay of Crude Bacteriocin Extracts Under Varied Culture Conditions

Bacteriocin PlnJK obtained from each of the 12 cultivation conditions was reconstituted to a concentration of 1 mg/mL. Its antibacterial activity against *S. enterica* ATCC 13076 was assessed using the double-layer agar diffusion assay. Briefly, 100 µL of the bacteriocin solution was loaded into wells punched in agar plates previously seeded with the indicator strain. After incubation, the diameters of the inhibition zones were measured with a digital vernier caliper to a precision of 0.01 mm.

Since the bacteriocin concentration and loading volume were consistent across samples, the diameter of the inhibition zone served as a direct proxy for the bacteriocin production level of strain EL2. Based on the comparative activity results from the 12 culture conditions, three representative sets were selected to establish defined cultivation models, corresponding to non-inducing, weakly inducing, and strongly inducing conditions for bacteriocin synthesis.

### 2.4. Screening for Optimal Inducing Strains

Fifteen safe strains, phylogenetically distinct from EL2, were selected as candidate inducers for co-culture screening. Each candidate strain was pre-cultured in its appropriate liquid medium through two sequential passages. Third-passage cultures were then used for co-culture experiments under the three predefined cultivation models (non-inducing, weakly inducing, and strongly inducing conditions for bacteriocin production). EL2 and each inducer were inoculated at an equal-volume ratio (1:1) into MRS broth and incubated for 24 h to obtain co-culture broths. For comparison, EL2 was also cultured alone in MRS broth for 24 h under the same three models to generate pure-culture controls. In both pure- and co-culture systems, the total inoculation volume was maintained at 2% (*v*/*v*).

CFS was prepared from both the pure-culture and co-culture broths, followed by partial purification of bacteriocin. The antibacterial activity of the resulting extracts (adjusted to 1 mg/mL) was determined against *S. enterica* ATCC 13076 via the agar diffusion assay, with inhibition zone diameters measured as described previously. For each of the three cultivation models, the difference in zone diameter between the co-culture and its corresponding pure-culture control was calculated. A positive difference served as the indicator of inducing capability, whereas a difference of zero or negative indicated no induction.

### 2.5. Susceptibility of Inducing Strains to Bacteriocin PlnJK

The inhibitory activity of bacteriocin PlnJK against each of the 15 candidate co-culture strains was assessed by measuring the diameter of the inhibition zones. All assays were conducted in triplicate, and the mean zone diameter was calculated for each strain.

### 2.6. Characterization of Co-Culture Derived Inducing Substances

CFS was prepared from the fermentation broth of the optimal inducing strain, *E. faecalis* MH2. To neutralize acidic components, the CFS was adjusted to pH 7.0 using NaOH. Catalase was then added to remove any residual hydrogen peroxide. The antibacterial activity of the treated CFS against *S. enterica* was subsequently evaluated by the agar diffusion assay, with inhibition zone diameters serving as the readout.

Crude bacteriocin extracts were prepared from eight distinct co-culture fermentation broths, each subjected to a different pre-treatment regimen. The antibacterial activity of each extract was then assessed by measuring the diameter of the corresponding inhibition zone (Table 2).

The Transwell co-culture system prevents direct physical contact between strains, thereby enabling the investigation of intercellular communication mediated by metabolic compounds.

### 2.7. Monitoring of Bacterial Growth and Bacteriocin Activity Kinetics over 24 h

*L. plantarum* EL2 and *E. faecalis* MH2 were pre-cultured through two sequential passages. They were then co-inoculated at a 1:1 (*v*/*v*) ratio into fresh MRS broth, achieving a total inoculum of 2% (*v*/*v*). A pure culture of EL2 alone was prepared in parallel as the control. The optical density at 600 nm (OD_600_) of both the pure-culture and co-culture broths was monitored at 2 h intervals over 24 h to generate growth curves [27].

In parallel, samples were collected from both the pure-culture and co-culture broths at 2 h intervals. Each sample was centrifuged, and crude bacteriocin extracts were prepared. The antibacterial activity of these extracts was assessed by measuring the inhibition zone diameter against *S. enterica* ATCC 13076.

### 2.8. Monitoring of AI-2 Autoinducer Concentration During a 24 h Period in Pure- and Co-Culture Systems

Fermentation broth samples were collected from both co-culture and pure-culture systems at 2 h intervals. The AI-2 autoinducer concentration in each sample was quantified using the bioluminescent reporter strain *V. harveyi* BB170, following established protocols [28].

### 2.9. Co-Culture-Induced Bacteriocin Synthesis: Condition Optimization

To optimize bacteriocin production, a stepwise screening of cultivation parameters was initiated. First, the co-culture performance of EL2 and MH2 was evaluated in five media: Potato Dextrose Broth (PDB), Brain Heart Infusion (BHI), Bacteriocin Production Medium (BPM), Tryptic Soy Broth (TSB), and de Man, Rogosa and Sharpe (MRS) broth. Subsequently, using MRS medium, suitable ranges for incubation time (8–24 h), temperature (34–38 °C), and initial pH (4.0–8.0) were determined. Based on these preliminary results, cell-associated parameters were further optimized. This included assessing the effects of different initial cell densities (10^4^–10^8^ CFU/mL) and the EL2:MH2 inoculation ratio (1:6, 1:3, 1:1, 3:1, 6:1) on bacteriocin yield. All optimization steps were assessed by measuring the inhibition zone diameter of the corresponding crude bacteriocin extract.

### 2.10. Optimization of Co-Culture Conditions via Response Surface Methodology

Based on preliminary single-factor experiments, three key factors—initial cell density (A), inoculation ratio (B), and initial pH (C)—were selected for response surface optimization. Each factor was assigned three levels, and a Box–Behnken design (BBD) was generated using Design-Expert^®^ software (v. 8.0.6.1). The inhibition zone diameter served as the response variable to model and identify the optimal cultivation conditions, following the methodology outlined by [29].

A Box–Behnken design was used to analyze the main and interactive effects of the selected factors on antibacterial activity via multiple regression modeling, with the aim of determining the optimal co-culture conditions. Verification experiments were performed in triplicate under the predicted optimum. Model validity was evaluated by comparing the experimentally measured antibacterial activity with the corresponding values predicted by the regression model (Table 3).

### 2.11. Minimum Inhibitory Doses (MIDs) of the Crude Bacteriocin Extract from Strain EL2 Against S. enterica Before and After Response Surface Optimization

#### 2.11.1. MID of the Crude Bacteriocin Extract from Strain EL2 Against *S. enterica* Under Pure Culture Conditions

A *S. enterica* suspension was harvested during the logarithmic growth phase and adjusted to 10^6^ CFU/mL. A 1.024 g sample of the crude bacteriocin extract (lyophilized powder obtained by ammonium sulfate precipitation, without further purification) was dissolved in 1 L of PBS to obtain a 1.024 mg/mL stock solution, which was then serially diluted twofold with PBS. Aliquots (100 μL) of the bacterial suspension and of the crude bacteriocin extract at various concentrations were added to the wells of a 96-well plate. After incubation at 37 °C for 16 h, the optical density at 600 nm (OD_600_) was measured. The lowest dose of the crude extract that completely inhibited the growth of *S. enterica* was defined as the MID. A mixture of 100 μL of the bacteriocin solution and 100 μL of sterile LB broth served as the negative control, while 200 μL of the bacterial suspension alone served as the positive control.

#### 2.11.2. MID of the Crude Bacteriocin Extract from Strain EL2 Against *S. enterica* Under Co-Culture Conditions

The MID of the crude bacteriocin extract obtained from the co-culture of strains EL2 and MH2 was determined against *S. enterica* using the method described in Section 2.11.1.

#### 2.11.3. MID of the Crude Bacteriocin Extract from Optimized Co-Culture (Strains EL2 and MH2) Against *S. enterica*

The MID of the crude bacteriocin extract obtained under optimized co-culture conditions was determined against *S. enterica* using the method described in Section 2.11.1.

### 2.12. Quantitative Analysis of Quorum-Sensing-Related Gene Expression

Gene sequences of *L. plantarum* were retrieved from the NCBI database. Primers were designed using Primer Premier 5.0 and commercially synthesized (Tsingke Biotechnology Co., Ltd., Beijing, China). The primer sequences are provided in Table 4.

Cell pellets were harvested from the pure culture (EL2) and co-culture (EL2 and MH2) groups at the late logarithmic phase (12 h and 14 h) and mid-stationary phase (18 h and 20 h), with three biological replicates per group at each time point. Total RNA was extracted from the harvested cell pellets using a commercial RNA extraction kit, and genomic DNA was removed by DNase digestion. Using total RNA as a template, cDNA was synthesized by reverse transcription with the Tsingke SynScript^®^ III RT SuperMix for qPCR. The resulting cDNA was diluted threefold and used as a template for qPCR amplification with the Tsingke ArtiCanCEO SYBR qPCR Mix. Following the manufacturer’s instructions, a 20 μL real-time quantitative PCR reaction mixture was prepared, with three technical replicates for each sample. The thermal cycling conditions were as follows: initial denaturation at 95 °C for 5 min, followed by 40 cycles of 95 °C for 15 s, 60 °C for 20 s, and 72 °C for 20 s.

The relative expression levels of the key QS gene *luxS* and the bacteriocin synthesis genes *plnA*, *plnB*, *plnC*, *plnD*, *plnJ*, and *plnK* were determined in the co-culture system at different time points using the 2^(−ΔΔCt) method, with *16S rRNA* as the reference gene and pure culture of EL2 during the logarithmic phase as the calibrator (expression level = 1).

### 2.13. Statistical Analysis

All experiments were conducted with at least three independent replicates, and the data are presented as the mean ± standard deviation (SD). Differences between groups were assessed using one-way analysis of variance (ANOVA). Statistical analyses were performed using SPSS software (version 19.0, IBM Corp., Armonk, NY, USA). Graphs were generated using OriginPro software (version 2019b, OriginLab Corporation, Northampton, MA, USA).

## 3. Results and Analysis

### 3.1. Design of Culture Systems for Non, Low, and Normal Bacteriocin Production

**Figure 1:**
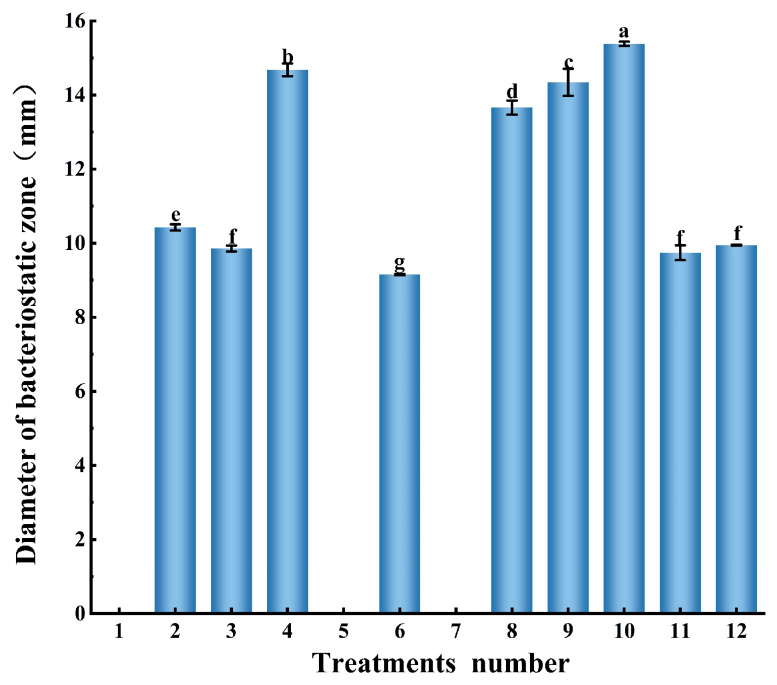
Bacteriocin yield/activity of *L. plantarum* EL2 under varying cultivation conditions. Different lowercase letters within the same column indicate significant differences at *p* < 0.05.

As shown in Figure 1 and Table 1, *L. plantarum* EL2 did not produce detectable bacteriocin under three of the twelve culture conditions (Groups 1, 5, and 7). Bacteriocin production remained low under five conditions (Groups 2, 3, 6, 11, and 12), whereas four conditions (Groups 4, 8, 9, and 10) supported robust synthesis. Based on these distinct yield profiles, three representative cultivation models were defined for subsequent studies: the non-producing model (Group 1: 33 °C, initial pH 5.0, 1% inoculum), the low-producing model (Group 6: 41 °C, initial pH 6.5, 1% inoculum), and the normal-producing model (Group 10: 37 °C, initial pH 6.5, 2.5% inoculum).

### 3.2. Screening for Optimal Inducing Strains

Among the 15 strains tested in co-culture, 10 exhibited an inductive effect on bacteriocin synthesis by EL2, 9 of which were lactic acid bacteria, similar to EL2. Both *L. plantarum* EA3 and CD11 exhibited relatively weak inductive effects. This suggests that microorganisms of the same species may exhibit weaker inductive effects due to competition for specific growth factors, whereas closely related microorganisms may possess greater inductive potential. The difference in inhibition zone diameter between co-culture and pure culture was negative for the remaining five strains co-cultured with EL2, indicating that these strains—including *Bacillus* spp. and *S. cerevisiae* (Table 5)—inhibited bacteriocin synthesis by EL2, possibly due to metabolites they produce that suppress EL2 growth. Eight of the inducing strains restored the ability of EL2 to synthesize bacteriocin under extreme conditions in which it failed to do so in pure culture, demonstrating that microbial co-culture can help strains adapt to the environment and enhance bacteriocin production. *E. faecium* MH2 exhibited the strongest inductive effect, increasing the inhibition zone diameter by 11.18 mm, 9.33 mm, and 10.19 mm under non-producing, low-producing, and normal-producing conditions, respectively.

### 3.3. Sensitivity of Co-Cultured Bacterial Strains to Bacteriocin Produced by L. plantarum EL2

Of the 15 strains tested, eight were sensitive to the bacteriocin PlnJK produced by EL2. These sensitive strains included *E. faecium*, *L. plantarum*, and *Bacillus* spp. In contrast, the remaining lactic acid bacteria and yeasts were insensitive. This result indicates that PlnJK exhibits a narrow inhibitory spectrum, with activity primarily against strains from the same genus (Table 6). Notably, *E. faecalis* MH2, the most potent inducer identified earlier, displayed only weak sensitivity to PlnJK. Another strong inducer, *L. fermentum* MK12, was completely insensitive. Although both *Bacillus* spp. and *S. cerevisiae* inhibited EL2’s bacteriocin production, their sensitivity to PlnJK differed markedly. Collectively, these results demonstrate that the inducing effect is highly strain-specific. Furthermore, no clear correlation was observed between a strain’s capacity to induce bacteriocin production in EL2 and its sensitivity to PlnJK.

### 3.4. Characterization of Co-Culture Derived Inducing Substances

**Figure 2:**
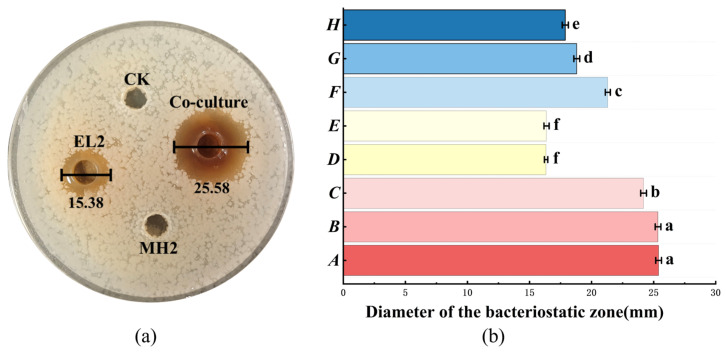
Induction capacity of different inducing substances on bacteriocin production by *L. plantarum* EL2. (**a**) Assessment of the antibacterial activity of candidate substances; (**b**) Identification and evaluation of key bacteriocin-inducing substances. The designations A through H (Table 2) in panel (**b**) correspond to specific inducing substances as detailed in Section 2.7.

Compared to pure culture, co-culture with MH2 significantly increased the diameter of the bacteriocin inhibition zone from 15.38 mm to 25.58 mm, representing a 1.66-fold enhancement in antibacterial activity. The CFS of MH2, following neutralization of acids and elimination of H_2_O_2_, did not produce an inhibition zone against *S. enterica* (Figure 2a). This indicates that MH2 itself does not produce bacteriocins or other direct antibacterial agents, but secretes specific compounds that induce bacteriocin production in EL2. No significant difference (*p* > 0.05) was observed between the inhibition zones produced in TransWell-separated co-culture and direct mixed co-culture (Figure 2b), suggesting that direct cell-to-cell contact is not required for the inducing effect. Although treated MH2 fermentation broth retained partial inducing activity, this activity was significantly lower than that observed in the normal co-culture system (*p* < 0.05). Among all treatments, the inducing capacity of the MH2 cell suspension most closely approximated that of the normal co-culture, indicating that metabolites from viable MH2 cells play a predominant role in the induction process.

### 3.5. Effect of Inducer Cell Density on Bacteriocin Production by L. plantarum EL2

**Figure 3:**
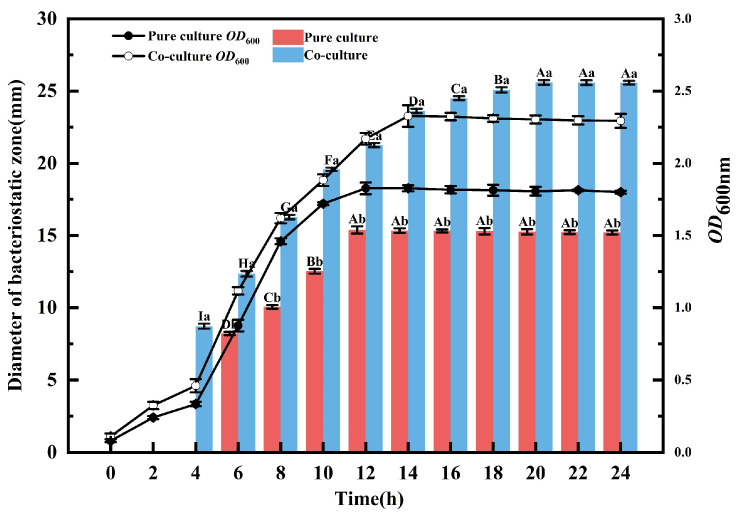
Dynamics of cell density and bacteriocin production during 24 h co-culture incubation. (a, b: Different lowercase letters within the same column indicate significant differences at *p* < 0.05. A, B, C, D, E, F, G, H, I: significant differences between pure culture groups or between co-culture groups at different time points).

The growth curves demonstrate distinct growth dynamics between the pure-culture and co-culture groups. Throughout the 24 h incubation, the cell density in co-culture consistently exceeded that in pure culture, achieving a maximum density 1.27-fold higher. While the pure-culture entered the exponential phase within 0–12 h and transitioned to stationary phase thereafter, the co-culture delayed entry into the stationary phase until 14 h (Figure 3). No bacteriocin was detected in either group during the first 2 h of incubation. Bacteriocin synthesis commenced earlier in co-culture (4 h) than in pure culture (6 h), indicating that co-culture advanced the onset of production. From 4 to 24 h, bacteriocin yield was consistently and significantly elevated in co-culture compared to pure culture (*p* < 0.01). Peak bacteriocin production occurred at 12 h in pure culture and at 20 h in co-culture. This demonstrates that co-culture not only enhances the yield but also extends the production phase. In both systems, the trends in cell density paralleled those of bacteriocin synthesis. This correlation confirms the significant influence of cell density on production, consistent with a cell-density-dependent regulation model for bacteriocin synthesis.

### 3.6. Dynamics of AI-2 Autoinducer Concentration in L. plantarum EL2 Pure-Culture and Co-Culture with E. faecalis MH2

**Figure 4:**
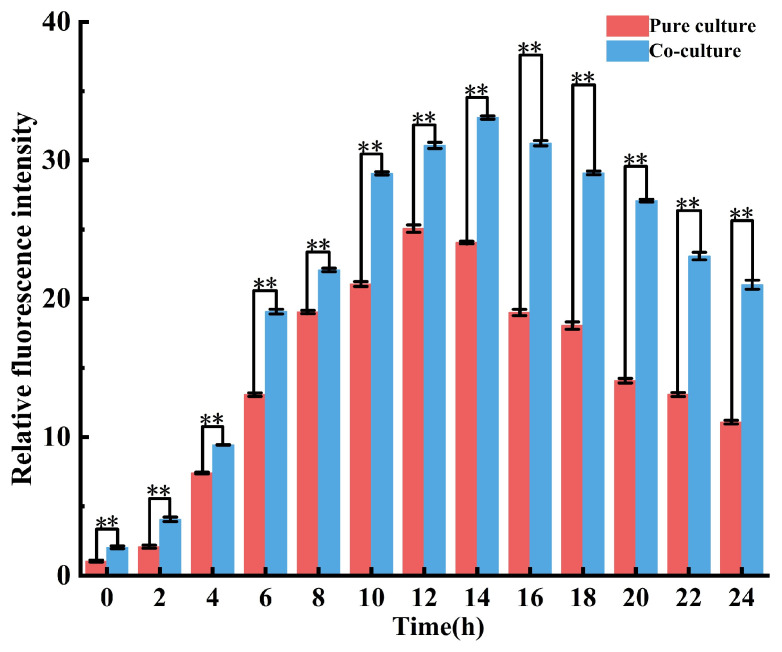
Concentration of the AI 2 autoinducer in *L. plantarum* EL2 pure culture and in co culture with *E. faecium* MH2 during 24 h incubation (** indicates a statistically significant difference compared to the pure culture group. (*p* < 0.01); Fermentation broth samples were collected from both co-culture and pure-culture systems at 2 h intervals. The AI-2 autoinducer concentration in each sample was quantified using the bioluminescent reporter strain *V. harveyi* BB170, following established protocols).

As shown in Figure 4, the AI-2 autoinducer concentration in both cultivation systems rose steadily from the initial lag phase through the exponential growth phase, peaked at the late-exponential phase, and subsequently declined gradually during the stationary phase. Throughout the incubation period, the AI-2 concentration in co-culture remained significantly higher than in pure culture, with a maximum relative fluorescence intensity 1.5-fold higher than that of the pure-culture control. These results demonstrate that co-culture significantly enhanced the production of the AI-2 signaling molecule (*p* < 0.01), which is consistent with its role in promoting bacteriocin synthesis.

### 3.7. Single-Factor Optimization of Culture Conditions for Bacteriocin Synthesis in Co-Culture

**Figure 5:**
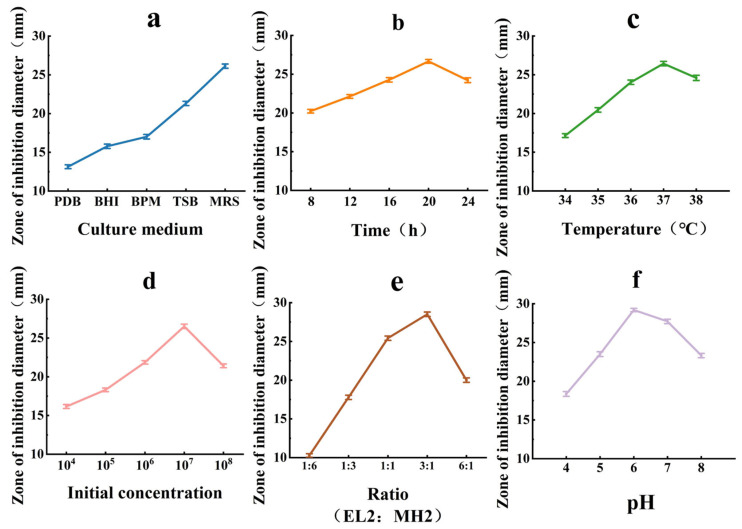
Effects of various cultivation conditions on bacteriocin synthesis in the *L. plantarum* EL2 and *E. faecalis* MH2 co-culture system. ((**a**): Effect of different culture media in co-culture on the diameter of the inhibition zone; (**b**): Effect of co-culture time on the diameter of the inhibition zone; (**c**): Effect of co-culture temperature on the diameter of the inhibition zone; (**d**): Effect of initial inoculum concentration on the diameter of the inhibition zone; (**e**): Effect of the ratio of EL2 to MH2 on the diameter of the inhibition zone; (**f**): Effect of co-culture pH on the diameter of the inhibition zone).

As shown in Figure 5, each factor (Culture medium, Time, Temperature, Initial concentration, Ratio, pH) exhibited a distinct effect on bacteriocin synthesis. Among the five media tested, MRS was the most favorable for bacteriocin synthesis (*p* < 0.05) and was therefore selected for subsequent experiments (Figure 5a). The inhibition zone diameter increased with co-culture time, although the rate of increase gradually diminished; it reached a maximum at 20 h and then decreased marginally. Consequently, 20 h was identified as the optimal co-culture time (*p* < 0.05) (Figure 5b). As the temperature increased, the inhibition zone diameter gradually enlarged, reaching a maximum at 37 °C (*p* < 0.05). Further increases in temperature led to reduced synthesis; therefore, 37 °C was selected as the optimal temperature (Figure 5c). Bacteriocin production increased with initial inoculum concentration, peaking at 10^7^ CFU/mL (*p* < 0.05), after which it was potentially inhibited by nutrient competition and the accumulation of metabolic by-products (Figure 5d). The maximum inhibition zone diameter (27.51 mm) was achieved at an inoculation volume ratio of 3:1 (EL2:MH2); an excessively high proportion of MH2 significantly inhibited EL2 activity (*p* < 0.01), indicating that this ratio provided the strongest inductive effect (Figure 5e). The inhibition zone diameter was largest at pH 6.0 (*p* < 0.05), while a decreasing trend was observed at pH 7.0 (Figure 5f). Thus, an initial pH of 6.0 was determined to be optimal.

### 3.8. Optimization of Co-Culture Conditions Using Response Surface Methodology

#### 3.8.1. Optimization of Co-Culture Conditions Using a Box–Behnken Design (BBD) and Corresponding Experimental Results

Single-factor experiments identified initial cell concentration, inoculation ratio, and initial pH as significant factors influencing bacteriocin synthesis. In contrast, cultivation temperature (37 °C) aligned with the conventional optimum, and extending the incubation time beyond 20 h yielded no further benefit. Consequently, these two factors were excluded from further optimization. Thus, three key variables were selected for response surface methodology (RSM): initial cell concentration (A), inoculation ratio (B), and initial pH (C). A BBD was employed to model the relationship between these variables, with inhibition zone diameter as the response variable, to predict the optimal culture conditions. A total of 17 experimental runs were designed according to the BBD matrix, and the corresponding results are presented in Table 7.

#### 3.8.2. Analysis of Variance (ANOVA) for the Box–Behnken Experimental Design

Based on the results of multiple regression analysis, a quadratic polynomial model was established to describe the relationship between the inhibition zone diameter (Y, response) and the three independent variables: initial cell concentration (A), inoculation ratio (B), and initial pH (C). The model is expressed by the following equation: Y = 30.166 + 1.04125A − 1.26875B − 0.24C − 1.55AB − 0.2075AC + 0.4575BC − 2.168A^2^ − 4.998B^2^ − 2.7405C^2^

Analysis of variance (ANOVA) revealed that the quadratic model was highly significant (F-value = 675.07, *p* < 0.0001; Table 8). The high coefficients of determination (R^2^ = 0.9988) and adjusted R^2^ (R^2^_Adj_ = 0.9974) indicated an excellent fit between the model predictions and the experimental data. The low coefficient of variation (C.V. = 0.7193%) further confirmed the high reproducibility of the experiments. All model terms—linear, interaction, and quadratic—exerted a highly significant influence on the response (*p* < 0.01). The relative influence of the factors, in descending order, was as follows: inoculation ratio > initial concentration > initial pH. Solving the regression equation yielded the following optimal conditions: initial cell concentration, 10^7.307^ CFU/mL; inoculation ratio, 3.056:1 (EL2:MH2); and initial pH, 5.929. Under these optimized conditions, the model predicted a maximum inhibition zone diameter of 30.45 mm.

#### 3.8.3. Analysis of Interaction Effects Among Factors

**Figure 6:**
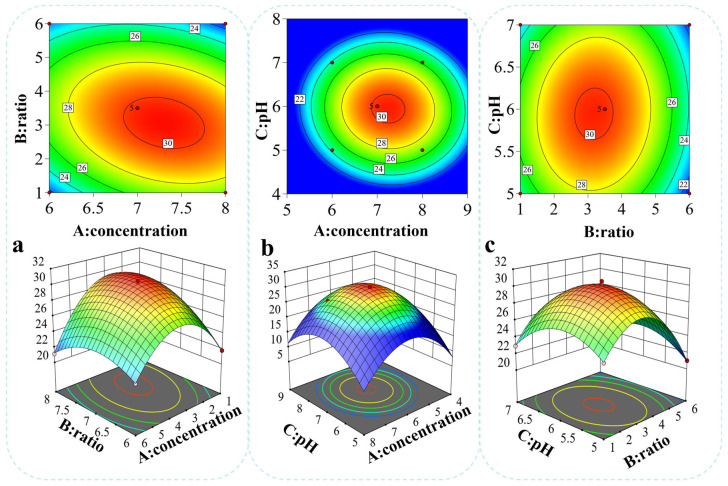
Response surface plots for the optimization of bacteriocin production in co-culture. ((**a**): Contour plot and three-dimensional response surface plot of the inhibition zone diameter under the interaction between initial inoculum concentration and co-culture ratio; (**b**): Contour plot and three-dimensional response surface plot of the inhibition zone diameter under the interaction between initial inoculum concentration and culture pH; (**c**): Contour plot and three-dimensional response surface plot of the inhibition zone diameter under the interaction between co-culture ratio and culture pH).

To visualize the interactive effects of the three factors (A, B, and C) on the inhibition zone diameter, contour plots and three-dimensional response surface plots were generated for each factor pair (Figure 6). The contour lines for the interaction terms AB and BC were elliptical and closely spaced, indicating that the interactions between initial inoculum concentration and co-culture ratio, as well as between pH and co-culture ratio, significantly affected the inhibition zone diameter (*p* < 0.01). In contrast, the contour lines for the interaction term AC were circular and widely spaced, suggesting that the interaction between initial inoculum concentration and culture pH had a minor effect on the inhibition zone diameter and could therefore be considered a secondary factor.

#### 3.8.4. Verification Experiment

The model predicted the following optimum conditions: an initial cell concentration of 10^7.307^ CFU/mL, an inoculation ratio of 3.056:1 (EL2:MH2), and an initial pH of 5.929, corresponding to a predicted inhibition zone diameter of 30.45 mm. For practical application, these conditions were rounded to feasible values: an initial concentration of 10^7^ CFU/mL, a 3:1 inoculation ratio, and an initial pH of 6.0. The experimentally measured inhibition zone diameter under these practical conditions was 30.32 mm, which closely aligned with the model prediction, thereby validating the model’s reliability. This final yield represents a 19% increase over the pre-optimization co-culture result (25.58 mm) and a 97% enhancement compared to the original pure-culture system, conclusively demonstrating the significant effectiveness of the co-culture optimization strategy.

### 3.9. MID of the Crude Bacteriocin Extract from Strain EL2 Against S. cerevisiae Before and After Response Surface Optimization

The optical density at 600 nm (OD_600_) of *S. enterica* decreased with increasing concentrations of the crude bacteriocin extract, indicating a negative correlation between the antibacterial effect and the extract concentration. When the concentration of the crude bacteriocin extract reached 256 μg/mL (Table 9), the OD_600_ value of *S. cerevisiae* decreased sharply and exhibited no significant difference from that of the negative control group (*p* > 0.05), demonstrating that the growth of *S. cerevisiae* was completely inhibited. Therefore, the MID of the crude bacteriocin extract from strain EL2 under pure culture conditions against *S. cerevisiae* was 256 μg/mL. Similarly, the MID of the crude bacteriocin extract obtained from the co-culture of strains EL2 and MH2 against *S. cerevisiae* was 64 μg/mL (Table 10), and that of the extract from the optimized co-culture was 32 μg/mL (Table 11). These results demonstrate that co-culture and subsequent optimization of culture conditions significantly enhanced the antibacterial activity of the crude bacteriocin extract, indicating a substantial increase in the concentration of active bacteriocin proteins, thereby reducing production costs.

### 3.10. Molecular Effect of Co-Culture on the Quorum-Sensing System and Bacteriocin Synthesis Genes

**Figure 7:**
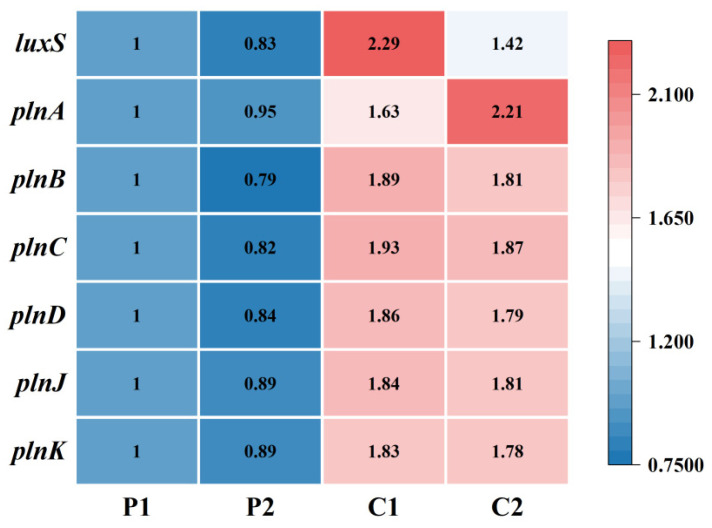
Relative expression levels of key genes for QS and bacteriocin synthesis. P1: logarithmic phase in pure culture. P2: stationary phase in pure culture. C1: logarithmic phase in co-culture. C2: stationary phase in co-culture.

To evaluate the effect of co-culture on bacteriocin synthesis, the relative expression levels of key genes were determined in the pure culture group at stationary phase (18 h) and in the co-culture group at both late logarithmic (14 h) and stationary phases (20 h). Gene expression in the pure culture group at late logarithmic phase (12 h) served as the calibrator (expression level = 1), with *16S rRNA* as the reference gene. The results showed that in the pure culture group, the expression of all related genes decreased during the stationary phase—a period characterized by reduced cell density and metabolic activity—indicating that bacteriocin synthesis is highly dependent on QS (Figure 7).

In the co-culture group, the expression levels of the *luxS* gene and genes related to bacteriocin synthesis were significantly higher than those in the pure-culture group during both the exponential and stationary phases. Specifically, the relative expression of the *luxS* gene (encoding the AI-2 autoinducer) was 2.29 during the exponential phase and was down-regulated to 1.42 in the stationary phase. In contrast, expression of the *plnA* gene, which encodes the AIP (autoinducing peptide) signal molecule, was higher during the stationary phase than during the exponential phase. Expression of genes involved in signal transduction and response (*plnB*, *plnC*, *plnD*) decreased in the stationary phase relative to the exponential phase. In contrast, the expression levels of the structural genes *plnJ* and *plnK*, which directly encode bacteriocin peptides, remained relatively stable across both growth phases (Table 8).

## 4. Discussion

Previous studies indicate that microbial co-cultures can establish stable interactions, enhancing resilience against environmental stressors that otherwise impair growth and metabolism [30]. Co-cultivation can improve strain adaptability, thereby supporting enhanced metabolic output even under suboptimal cultivation parameters [15]. In this work, we established defined cultivation models that simulate extreme environments to challenge lactic acid bacteria growth. Microbial co-culture significantly enhanced the tolerance of *L. plantarum* EL2 to these stressors. Notably, eight inducing strains enabled bacteriocin synthesis under conditions that completely abolished production in monoculture. The most potent inducer, *E. faecalis* MH2, increased the yield of bacteriocin PlnJK by approximately 1.66-fold. Subsequent optimization of the co-culture conditions further increased PlnJK production by approximately 1.97-fold. This aligns with reports such as that of Ye et al., who achieved a substantial increase in bacteriocin Paracin 1.7 yield via co-culture optimization [14]. This approach thus represents a sustainable and economically favorable strategy for process intensification and high-titer bacteriocin production in industrial lactic acid bacteria fermentations.

The Transwell co-culture system physically separates the strains, eliminating direct cell-to-cell contact and allowing the role of extracellular metabolites in intercellular communication to be specifically investigated [31,32]. The bacteriocin produced in the Transwell system was identical to that synthesized in direct co-culture, demonstrating that direct physical contact is not a prerequisite for induction and that informational exchange is primarily mediated by diffusible extracellular metabolites [33]. The *luxS* protein, encoded by the *luxS* gene, is a known key mediator in co-culture-induced bacteriocin synthesis and is susceptible to degradation by proteinase K [34]. However, the CFS of MH2 retained partial inducing activity following heat inactivation or proteinase K treatment. This suggests that a component of the inducing factors produced by MH2 is not proteinaceous in nature.

The enhanced bacteriocin synthesis observed in the co-culture system is dependent on the AI-2 autoinducer, whose production is mediated by the *luxS* gene and which is subject to gradual inactivation and degradation over the cultivation period [35,36]. Within co-culture systems, the autoinducing peptide (AIP, mediated by *plnA*) and AI-2 (mediated by *luxS*) have been shown to independently promote bacteriocin synthesis [21]. In the EL2 and MH2 co-culture, AI-2 concentration began to decline in the late exponential phase (14 h), whereas bacteriocin production continued to increase. This indicates that residual, active inducing substances persisted in the system beyond 14 h. RT-qPCR analysis revealed distinct temporal expression patterns: *luxS* gene expression was significantly elevated during the exponential phase compared to the stationary phase, while *plnA* expression exhibited an inverse pattern, being significantly lower during exponential growth. In contrast, the expression levels of genes encoding the three-component regulatory system (*plnB*, *plnC*, *plnD*) and the bacteriocin structural genes (*plnJ*, *plnK*) remained relatively stable across both growth phases and were consistently and significantly higher than those in the pure-culture group. Collectively, these findings support a model wherein, during the exponential co-culture phase, accumulation of *luxS*-mediated AI-2 promotes efficient activation of the quorum-sensing circuitry, initiating a gradual increase in PlnJK synthesis [37]. In the subsequent stationary phase, the regulatory influence shifts, and AIP encoded by *plnA* becomes the predominant factor sustaining bacteriocin yield. This proposed temporal regulatory mechanism provides a theoretical framework for understanding how quorum-sensing systems orchestrate efficient bacteriocin synthesis in lactic acid bacteria [38].

The AI-2 autoinducer is chemically derived from 4,5-dihydroxy-2,3-pentanedione (DPD) and can be produced synthetically. Exogenously supplied synthetic AI-2 can significantly modulate the QS systems of lactic acid bacteria [39,40,41]. Certain metabolites, such as maltose or acetate, have been reported to stimulate *plnA* gene expression [42,43]. Exogenous AI-2 can therefore substitute for interspecies QS, inducing increased bacteriocin synthesis during the logarithmic growth phase. However, the signal molecule responsible for this effect during the stationary phase remains unidentified.

The inducing substances produced by MH2 that are independent of the *luxS*/AI-2 system only begin to influence EL2 during the stationary phase of co-culture. Consequently, the upregulation of the *plnA* gene during the co-culture stationary phase may be attributed to either (i) specific compounds produced by MH2 that promote bacteriocin metabolism in EL2, or (ii) the continuous accumulation of MH2-secreted metabolites, which creates chemical stress on EL2, thereby inducing it to release more bacteriocin as a self-protective response. For instance, He et al. reported that ascorbic acid, N-acetyl-L-glutamic acid, butyryl trihexyl citrate, N-acetyl aspartate, and L-gulonolactone enhanced bacteriocin synthesis in co-culture systems [32]. Among these, amino acid-type substances promoted the growth of lactic acid bacteria and increased the expression of genes related to bacteriocin metabolism, whereas butyryl trihexyl citrate inhibited the growth of lactic acid bacteria at elevated concentrations [37]. Therefore, precise control of the co-culture duration is critical to ensure that these metabolites remain at appropriate concentrations, thereby improving the stability of enhanced bacteriocin synthesis.

In this study, preliminary observations indicated that bacteriocin synthesis was induced by the co-culture system; however, the underlying molecular mechanism remains unclear. To further elucidate this regulatory process, our research group plans to employ targeted metabolomics and other approaches to systematically identify the key signaling molecules within the co-culture system, thereby clarifying the chemical nature of the bacteriocin induction. Building on this, we aim to achieve precise regulation of bacteriocin production via the exogenous addition of target inducers. This approach could provide a feasible strategy to mitigate yield fluctuations during fermentation and reduce production costs. These investigations will help elucidate the intrinsic relationship between microbial interactions and secondary metabolite regulation at the molecular level, thereby advancing the design of efficient bacteriocin production processes.

## 5. Conclusions

By employing a microbial co-culture strategy, this study substantially enhanced the environmental tolerance of the PlnJK-producing strain EL2, enabling it to maintain its biosynthetic capacity even under extreme culture conditions. Following optimization, MH2 was identified as the optimal inducing strain; under the optimized initial inoculum concentration and ratio, PlnJK production was increased by 1.97-fold. Further analysis revealed that both cell density and the concentration of the AI-2 signaling molecule were significantly higher in the co-culture system than in the pure culture, consistent with the density-dependent characteristics of QS. During the logarithmic phase of co-culture, the interspecific *luxS*/AI-2 system stimulated the expression of the *plnBCD* genes, thereby promoting bacteriocin synthesis. Upon entering the stationary phase, the accumulation of characteristic metabolites from MH2 exerted environmental stress on EL2, leading to the upregulation of the *plnA* gene encoding AIP and further enhancing PlnJK production. These findings clarify the timing and mode of action of AIP and AI-2 in the co-culture system, providing a novel perspective and a theoretical foundation for the QS system-mediated induction of efficient bacteriocin synthesis.

## Figures and Tables

**Table 1:** Design of a Gradient of Cultivation Conditions.

Treatments number	1	2	3	4	5	6	7	8	9	10	11	12
Inoculation concentration (%)	1	1	1	1	1	1	2.5	2.5	2.5	2.5	2.5	2.5
Temperature (°C)	33	33	37	37	41	41	33	33	37	37	41	41
Starting pH	5	6.5	5	6.5	5	6.5	5	6.5	5	6.5	5	6.5

**Table 2:** Culture fermentation broth with different treatments.

Group	Treatment
A	EL2 and MH2 were co-cultured directly in MRS broth under standard conditions
B	EL2 and MH2 were co-cultured in physical separation using a Transwell^®^ chamber system (Seville Biology, Wuhan, China) [26]
C	MH2 cells were harvested by centrifugation (10,000× *g*, 4 °C), washed three times with physiological saline, and resuspended
D	The washed MH2 cell suspension was mechanically disrupted to obtain a cell homogenate
E	The MH2 cell suspension was heat-inactivated (121 °C, 15 min)
F	CFS was prepared from MH2 fermentation broth via centrifugation
G	MH2 CFS was heat-inactivated (121 °C, 15 min)
H	MH2 CFS was treated with proteinase K (2 mg/mL, pH 8.0, 37 °C, 2 h), followed by enzyme inactivation (80 °C, 15 min)

The above-mentioned treatments from groups C to H were added to the culture of EL2 and incubated under the specified conditions.

**Table 3:** Experimental factors and their levels for the Box–Behnken design.

Factor	Level		
	−1	0	1
A: Initial concentration (lg value)	6	7	8
B: Ratio	1	3	6
C: Initial pH	5	6	7

**Table 4:** Primer sequences used in this study.

Gene Name	Direction	Nucleotide Sequence (5′→3′)	Product Size
*luxS*	F	CATGACACCGTTGAAGTTGC	103
	R	CACAGCTATCGATGGTCG	
*plnA*	F	CAACTCTGGTTCTGCAGGTG	110
	R	CGTAGTATAGTGGCTGTTGT	
*plnB*	F	CTTACGCCAATCTATTATACG	143
	R	GGTCTGCGTATAAGCATCGC	
*plnC*	F	CTATAGTGCCATGCTCTACG	116
	R	GCAGTATCGTATAGGTCGATA	
*plnD*	F	ACCGTTAGGTACAACGCTAGG	127
	R	CTACGGTACTGAACTCGGACG	
*plnJ*	F	AATCAAGGAATTATATTAGTC	103
	R	AATCGCAGTGACTTCCAGAAC	
*plnK*	F	AGAGCAATGTCGTTAATAAATG	129
	R	TATGATGAAAAAAATTGAAAA	
*16S rRNA*	F	CGGCGTGCTAATCATACAAGTC	114
	R	GAAGCCATCATTCAATCTCGG	

**Table 5:** Screening for bacterial strains that induce bacteriocin production by *L. plantarum* EL2 in co-culture under three distinct cultivation models (Quantification of the difference in inhibition zone diameter of bacteriocin PlnJK against *S. cerevisiae* before and after co-culture).

Co-Culture Strain	Difference in Diameter of Bacteriostatic Zone (mm)		
	No Yield Model	Low Yield Model	Normal Yield Model
*E. faecalis* MH2	11.18 ± 0.09	9.33 ± 0.12	10.01 ± 0.09
*L. fermentum* MK12	9.98 ± 0.12	8.23 ± 0.08	8.44 ± 0.12
*L. paracasei* AC13	9.64 ± 0.11	5.28 ± 0.05	7.36 ± 0.07
*E. faecium* S84	8.86 ± 0.07	4.51 ± 0.11	6.25 ± 0.15
*L. mesenteroides* SL2	9.15 ± 0.13	3.14 ± 0.14	5.93 ± 0.04
*E. durans* S12	9.35 ± 0.02	2.55 ± 0.09	3.61 ± 0.11
*L. lactis* SL1	8.25 ± 0.09	3.05 ± 0.06	3.17 ± 0.02
*P.pastoris* BK-11	8.23 ± 0.04	2.48 ± 0.02	2.86 ± 0.05
*L. plantarum* CD11	0	2.14 ± 0.05	2.05 ± 0.03
*L. plantarum* EA3	0	2.14 ± 0.03	1.98 ± 0.02
*B. subtilis* QSM-27	0	−0.93 ± 0.02	−1.20 ± 0.02
*B. subtilis* QSM-199	0	−0.98 ± 0.01	−1.24 ± 0.06
*B.pumilus* CF88	0	−9.15 ± 0.16	−4.60 ± 0.12
*S. cerevisiae* SD-7	0	−9.15 ± 0.17	−6.80 ± 0.08
*S. cerevisiae* SD-1	0	−9.15 ± 0.14	−15.38 ± 0.16

**Table 6:** Sensitivity of co cultured bacterial strains to bacteriocin PlnJK from *L. plantarum* EL2.

Strain	Diameter of the Bacteriostatic Zone (mm)	Antimicrobial Capacity
*E. faecalis* MH2	9.95 ± 0.07	+
*provid. fermentum* MK12	0.00	−
*L. paracasei* AC13	0.00	−
*E. faecium* S84	10.16 ± 0.16	+
*L. mesenteroides* SL2	0.00	−
*E. durans* S12	10.12 ± 0.17	+
*L. lactis* SL1	0.00	−
*P. pastoris* BK-11	0.00	−
*L. plantarum* CD11	13.76 ± 0.15	++
*L. plantarum* EA3	13.63 ± 0.14	++
*B. subtilis* QSM-27	17.87 ± 0.12	+++
*B. subtilis* QSM-199	16.92 ± 0.09	+++
*B. pumilus* CF88	16.61 ± 0.21	+++
*S. cerevisiae* SD-7	0.00	−
*S. cerevisiae* SD-1	0.00	−

+: with weak inducing ability; ++: with strong inducing ability; +++: with extremely strong inducing ability.

**Table 7:** Results of single-factor experiments optimizing bacteriocin production in co-culture.

Test No.	Initial Concentration (MH2 and EL2) (logCFU/mL) (A)	Ratio (Volume Ratio of EL2 to MH2) (B)	Initial pH (C)	Bactericidal Zone Diameter (mm)
1	7	3.5	6.0	30.35
2	7	3.5	6.0	30.01
3	8	6	6.0	21.12
4	6	3.5	5.0	24.21
5	7	3.5	6.0	30.09
6	6	3.5	7.0	24.15
7	6	1	6.0	21.78
8	8	3.5	5.0	26.78
9	7	6	7.0	21.44
10	8	3.5	7.0	25.89
11	6	6	6.0	22.21
12	7	6	5.0	21.01
13	7	1	5.0	24.33
14	7	3.5	6.0	30.07
15	8	1	6.0	26.89
16	7	1	7.0	22.93
17	7	3.5	6.0	30.11

**Table 8:** Analysis of variance (ANOVA) for the quadratic polynomial model.

Source	Sum of Squares	df	Mean Squares	F-Value	*p*-Value	Significance
Model	204.49	9	22.72	675.07	<0.0001	significant
A-Concentration	8.67	1	8.67	257.71	<0.0001	
B-Ratio	12.88	1	12.88	382.63	<0.0001	
C-pH	0.4608	1	0.4608	13.69	0.0077	
AB	9.61	1	9.61	285.53	<0.0001	
AC	0.1722	1	0.1722	5.12	0.0581	
BC	0.8372	1	0.8372	24.88	0.0016	
A^2^	19.79	1	19.79	588.01	<0.0001	
B^2^	105.18	1	105.18	3125.08	<0.0001	
C^2^	31.62	1	31.62	939.57	<0.0001	
Residual	0.2356	7	0.0337			
Lack of fit	0.0457	3	0.0152	0.3207	0.8114	not significant
Pure error	0.1899	4	0.0475			
Cor total	204.72	16				
R^2^	0.9988					
Adj-R^2^	0.9974					
Pre-R^2^	0.9950					
C.V. %	0.7193					

**Table 9:** The MID of the bacteriocin from strain EL2 was determined. (OD_600_: Optical density of the bacterial suspension at 600 nm, used to estimate bacterial cell density).

Bacteriocin Concentration (μg/mL)	Positive Control	0.25	0.5	1	2
OD_600nm_	0.583 ^a^	0.551 ^b^	0.509 ^c^	0.436 ^d^	0.387 ^e^
Bacteriocin concentration (μg/mL)	4	8	16	32	64
OD_600nm_	0.318 ^f^	0.257 ^g^	0.199 ^h^	0.142 ^i^	0.129 ^j^
Bacteriocin concentration (μg/mL)	128	256	512	1024	Negative control
OD_600nm_	0.101 ^k^	0.083 ^l^	0.82 ^l^	0.82 ^l^	0.082 ^l^

Different lowercase letters within the same column indicate significant differences at *p* < 0.05.

**Table 10:** The MID of the bacteriocin produced during co-culture of strains EL2 and MH2 was determined.

Bacteriocin Concentration (μg/mL)	Positive Control	0.25	0.5	1	2
OD_600nm_	0.583 ^a^	0.531 ^b^	0.493 ^c^	0.414 ^d^	0.362 ^e^
Bacteriocin concentration (μg/mL)	4	8	16	32	64
OD_600nm_	0.285 ^f^	0.201 ^g^	0.165 ^h^	0.099 ^i^	0.082 ^j^
Bacteriocin concentration (μg/mL)	128	256	512	1024	Negative control
OD_600nm_	0.082 ^j^	0.081 ^j^	0.081 ^j^	0.082 ^j^	0.082 ^j^

Different lowercase letters within the same column indicate significant differences at *p* < 0.05.

**Table 11:** The MID of the bacteriocin produced during co-culture of strains EL2 and MH2 following response surface optimization was determined.

Bacteriocin Concentration (μg/mL)	Positive Control	0.25	0.5	1	2
OD_600nm_	0.583 ^a^	0.471 ^b^	0.404 ^c^	0.339 ^d^	0.271 ^e^
Bacteriocin concentration (μg/mL)	4	8	16	32	64
OD_600nm_	0.203 ^f^	0.169 ^g^	0.115 ^h^	0.83 ^i^	0.082 ^i^
Bacteriocin concentration (μg/mL)	128	256	512	1024	Negative control
OD_600nm_	0.082 ^i^	0.082 ^i^	0.082 ^i^	0.082 ^i^	0.082 ^i^

Different lowercase letters within the same column indicate significant differences at *p* < 0.05.

## Data Availability

The original contributions presented in this study are included in the article. Further inquiries can be directed to the corresponding author.
